# SGK1 inhibition-induced autophagy impairs prostate cancer metastasis by reversing EMT

**DOI:** 10.1186/s13046-018-0743-1

**Published:** 2018-04-02

**Authors:** Weiwei Liu, Xuchu Wang, Yiyun Wang, Yibei Dai, Yiyi Xie, Ying Ping, Binbin Yin, Pan Yu, Zhenping Liu, Xiuzhi Duan, Zhaoping Liao, Yuhua Chen, Chunhua Liu, Xiang Li, Zhihua Tao

**Affiliations:** 1 Department of Laboratory Medicine, The Second Affiliated Hospital of Zhejiang University School of Medicine, Hangzhou, China; 2 Department of Blood Transfusion, The Second Affiliated Hospital of Zhejiang University School of Medicine, Hangzhou, China

**Keywords:** SGK1, Prostate cancer, Autophagy, EMT, Metastasis

## Abstract

**Background:**

Despite SGK1 has been identified and characterized as a tumor-promoting gene, the functions and underlying mechanisms of SGK1 involved in metastasis regulation have not yet been investigated in cancer.

**Methods:**

We investigated the cellular responses to GSK650394 treatment and SGK1 silencing (or overexpression) in human prostate cancer (PCa) cell lines and PC3 xenografts by wound healing assay, migration and invasion assay, western blotting, immunofluorescence and immunohistochemistry.

**Results:**

In the present study, we found that SGK1 expression positively correlates with human prostate cancer (PCa) progression and metastasis. We show that SGK1 inhibition significantly attenuates EMT and metastasis both in vitro and in vivo, whereas overexpression of SGK1 dramaticlly promoted the invasion and migration of PCa cells. Our further results suggest that SGK1 inhibition induced antimetastatic effects, at least partially via autophagy-mediated repression of EMT through the downregulation of Snail. Moreover, ectopic expression of SGK1 obviously attenuated the GSK650394-induced autophagy and antimetastatic effects. What’s more, dual inhibition of mTOR and SGK1 enhances autophagy and leads to synergistic antimetastatic effects on PCa cells.

**Conclusions:**

Taken together, this study unveils a novel mechanism in which SGK1 functions as a tumor metastasis-promoting gene and highlights how co-targeting SGK1 and autophagy restrains cancer progression due to the amplified antimetastatic effects.

**Electronic supplementary material:**

The online version of this article (10.1186/s13046-018-0743-1) contains supplementary material, which is available to authorized users.

## Background

Prostate cancer (PCa) remains the most common malignancy diagnosed in men and the second leading cause of male cancer-related deaths in the Western world [1]. Although the improvements in PCa diagnostic methods and in multiple treatments have led to a dramatic decrease in PCa-related deaths in the last three decades, and for patients in the United States who develop metastatic disease, the 5-year survival rate is only 29% [2]. Thus, it’s urgent to develop novel therapeutic strategies to combat cancer metastasis and prevent cancer progression.

It is widely accepted that the initial step, acquisition of migration and invasion capability, is the rate-limiting step in metastatic cascade [3]. Epithelial-mesenchymal transition (EMT) is proposed to be an important mechanism regulating the initial steps in cancer metastasis and progression [4]. EMT is a complex biological process that epithelial cells undergo reprogramming from a polarized, differentiated phenotype with numerous cell-cell junctions to obtain a mesenchymal phenotype including lack of polarization, decreased cell-cell junctions, increased motility [4]. In fact, this process is dynamic and plastic as the migratory cancer cells undergo the reverse process, termed mesenchymal-epithelial transition (MET), to recolonize and proliferate at distant metastatic sites [4–6]. The EMT/MET processes are regulated by a number of factors, among which the SNAI family members Snail and Slug are known to repress E-cadherin expression in epithelial cells undergoing EMT, but no evidences exist on their roles on other members of the cadherin family, neither additional roles on target genes [3, 7, 8].

Autophagy (also known as macroautophagy), or cellular self-digestion, is a highly conserved catabolic process that targets cellular contents to the lysosomal compartment for degradation, with an astonishing number of connections to human physiology and disease [9]. Emerging evidence shows that autophagy is upregulated during cellular stress, which has been demonstrated to suppress primary tumor formation [10, 11], but how autophagy influences metastasis remains unknown [12].

Serum- and glucocorticoid-induced protein kinase 1 (SGK1) belongs to the ‘AGC’ subfamily of protein kinases and shares approximately 54% identity of its catalytic domain with protein kinase B (PKB, also called Akt) [13]. SGK1 is identified and characterized as a tumor-promoting gene and elevated expression of SGK1 has been observed in several different malignancies, including colon cancer [14], gastric cancer [15] and prostate cancer [16]. Particularly, SGK1-overexpressing PCa xenografts displayed accelerated castrate-resistant tumor initiation, supporting a role for SGK1-mediated PCa progression [17]. In addition, HEK293 cells transiently transfected with the constitutively active SGK1 mutant plasmid acquires enhanced cell migration capacity via vinculin dephosphorylation [18]. Ablation of SGK1 impairs endothelial cell migration and tube formation leading to decreased neo-angiogenesis in vitro [19]. Collectively, these observations and findings suggest that SGK1 plays a significant role in metastasis. However, the functions and underlying mechanisms of SGK1 involved in invasion and metastasis regulation have not yet been investigated in cancer.

In this study, we investigated the functional significance of SGK1 in EMT and metastasis regulation in PCa. Our findings showed that SGK1 exhibited a significant upregulation in primary metastatic PCa tissues, and downregulation of SGK1 could induce autophagy, which contributes to suppress metastasis and reverse the EMT through the downregulation of Snail, whereas its overexpression could attenuate autophagic activity and promote the EMT and metastasis in PCa.

## Results

### SGK1 expression is elevated in primary metastatic PCa tissues

**Fig. 1:**
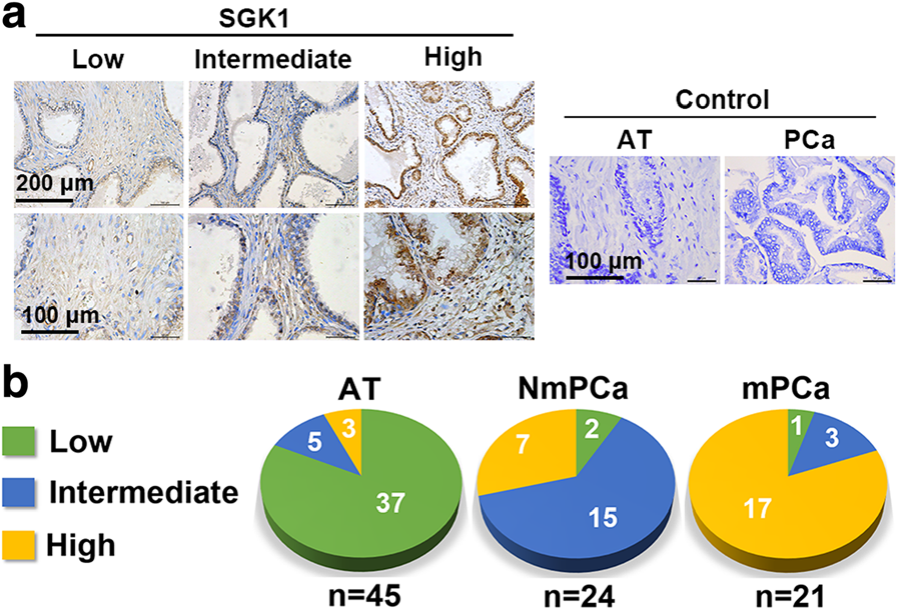
Immunohistochemical analysis for SGK1 in PCa tissues. **a** Representative images (including low, intermediate and high expression) from the immunohistochemical analysis for SGK1 in primary nonmetastatic PCa (NmPCa) tissues, primary metastatic PCa (mPCa) tissues and corresponding adjacent normal prostate tissues (AT). **b** Quantification of the stainings observed in 45 PCa cases

We first determined whether SGK1 expression is associated with human PCa progression. Immunohistochemistry staining was performed in 24 primary nonmetastatic PCa (NmPCa) and 21 primary metastatic PCa (mPCa) tissues, all of which contain tumor adjacent normal prostate tissues (AT) and PCa tissues. The representative pictures were shown in Fig. 1a. Our results showed that there was a significantly lower level of SGK1 expression in AT than in the NmPCa tissues, and SGK1 expression was the most intense in mPCa tissues (Fig. 1b). These suggested that SGK1 was commonly expressed in normal human prostate tissue but increased in PCa tissue, and implies that SGK1 expression may positively correlate with human PCa progression.

### SGK1 inhibition impairs PCa cells migration and invasion in vitro

**Fig. 2:**
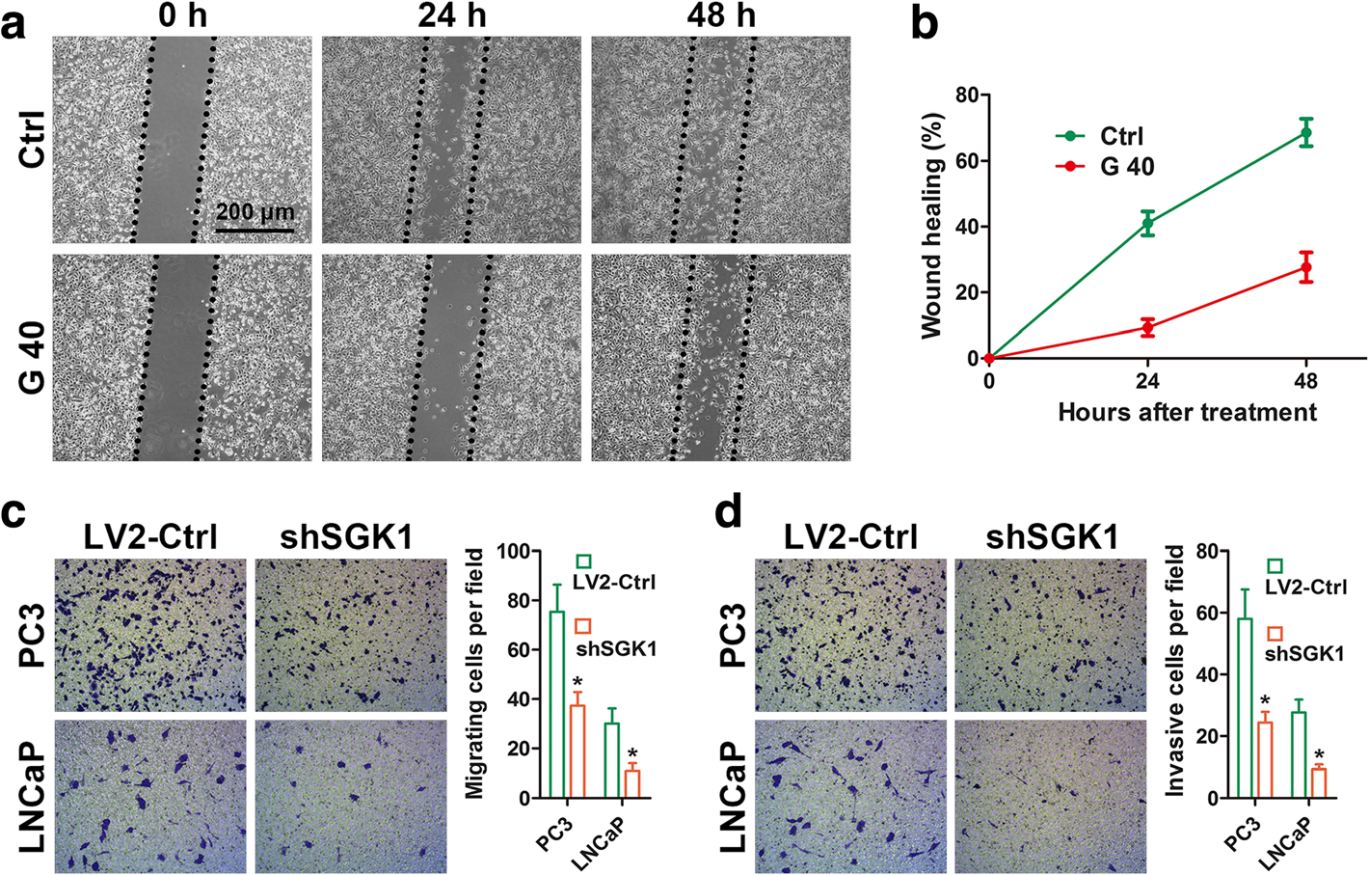
SGK1 inhibition impairs PCa cells migration and invasion in vitro. **a** Wound healing assays of PC3 cells treated with DMSO or 40 μM GSK650394. Phase-contrast images were acquired at 0, 24 and 48 h after scratching and representative images of three independent experiments are shown. The wound healing area was analyzed by using ImageJ software and the corresponding data, relative to 0 h, expressed in the graph (**b**). **c** Cell migration assays were performed on SGK1-depleted PC3 and LNCaP cells (shSGK1) and on control cells (LV2-Ctrl). Representative fields of migration cells on the membrane are shown (magnifications, × 200). Average migration cell number per field is quantified. **d** Matrigel cell invasion assays were performed as in **c**. Representative images show the cells that invaded through the matrigel. Representative histograph of invaded tumor cells is displayed and number of invaded tumor cells quantified. * indicates significant difference from the controls (* *P* < 0.05, ANOVA)

**Fig. 3:**
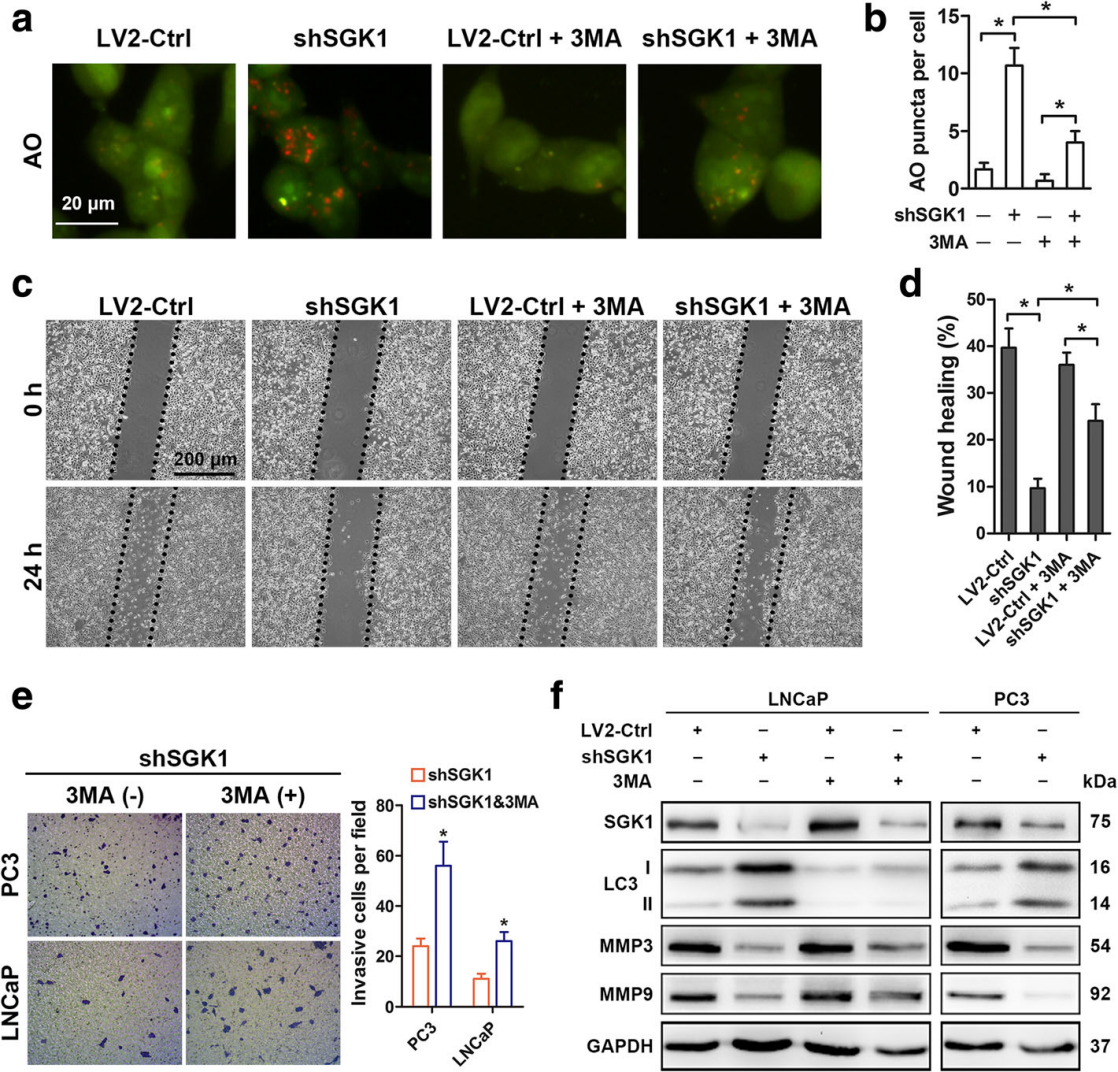
SGK1 inhibition induces autophagy, which contributes to metastasis suppression. **a-e** PC3 and LNCaP cells were infected with empty lentivirus control (LV2-Ctrl) or with the lentivirus SGK1-shRNA (LV2-shSGK1) and subsequently incubated for 24 h before pretreatment with 5 mM 3MA or PBS (control). Autophagy activity was determined with AO staining (**a**) and quantified (**b**) in LNCaP cells. **c** Wound healing assays of PC3 cells. The wound healing area was analyzed by using ImageJ software and the corresponding data, relative to 0 h, expressed in the graph (**d**). **e** Matrigel cell invasion assays were performed both in PC3 and LNCaP cells. Representative images show the cells that invaded through the matrigel. Representative histograph of invaded tumor cells is displayed and number of invaded tumor cells quantified. **f** PC3 and LNCaP cells were infected with empty lentivirus control (LV2-Ctrl) or with the lentivirus SGK1-shRNA (LV2-shSGK1) and subsequently incubated for 48 h before pretreatment with 5 mM 3MA or PBS (control). Western blots were probed for levels of SGK1, LC3-I, LC3-II, MMP3 and MMP9, and GAPDH was used as a loading control. All results are representative of three experiments and are expressed as the mean ± S.D.; *P < 0.05

Since SGK1 expression is related to PCa progression, SGK1 may play important roles in one or more steps of PCa metastasis. We first examine the effect of SGK1 inhibition on PCa cells migration and invasion. The migration capability of PCa cell lines was analyzed by means of a wound healing assay. We observed a strong inhibition of 40 μM GSK650394 (an inhibitor of SGK1) on PC3 cells, in comparison with control cells after 24 and 48 h of treatment (Fig. 2a and b), but it had almost no effect on PC3 cells viability (data shown in our previous study [20]). Similar phenomenon was also observed in DU145 cells after treatment with 20 μM GSK650394 (Additional file 1: Figure S1). We further confirmed these findings using a transwell migration assay, consistent with results obtained from wound healing assay, lentiviral shRNA-mediated SGK1 silencing, as confirmed by immunoblotting (Fig. 3f), was significantly decreased migration of PC3 and LNCaP cells after 24 h of treatment (Fig. 2c), but it had almost no effect on cells viability (data shown in our previous study [20]). Finally, the effect of SGK1 inhibition upon invasion was assessed by means of a transwell-based assay, in the presence of matrigel, as anticipated, SGK1 inhibition potently decreased invasion of both PC3 and LNCaP cells (Fig. 2d). In summary, SGK1 inhibition, mediated by either GSK650394 or shSGK1, significantly impairs migration and invasion of PCa cells.

### SGK1 inhibition induces autophagy, which contributes to metastasis suppression

A previous study has suggested that SGK1 is involved in the regulation of muscle mass maintenance through negatively regulating autophagy [21]. However, the relationship between SGK1 and autophagy has never been reported in PCa before, especially in the modulation of metastasis. In order to illuminate the function of SGK1 in the regulation of autophagy in PCa, first, autophagy activity was evaluated with AO staining after SGK1 elimination, as shown in Fig. 3a, shSGK1 exhibited a stark increase in discrete acidic vesicles compared to LV2-Ctrl in LNCaP cells (Fig. 3b), which was further confirmed by increased LC3II protein levels both in shSGK1-LNCaP and -PC3 cells (Fig. 3f). Thus, these results suggested that SGK1 inhibition significantly induced autophagy in PCa. Next, the role of SGK1 inhibition-induced autophagy in PCa metastasis was investigated. Our results showed that treatment with 3MA significantly decreased SGK1 inhibition-induced AO puncta (Fig. 3a and b) and SGK1 inhibition-dependent LC3-I to LC3-II conversion, as expected (Fig. 3f). Moreover, the inhibition of autophagy by 3MA antagonized the inhibitory effects of SGK1 inhibition on cell migration (Fig. 3c and d) and invasion (Fig. 3e), which was consistent with the increased MMP3 and MMP9 protein expression (Fig. 3f). Collectively, these results suggest that SGK1 silencing induces autophagy, which contributes to metastasis suppression.

### SGK1 overexpression attenuates autophagy and promotes cell migration and invasion

**Fig. 4:**
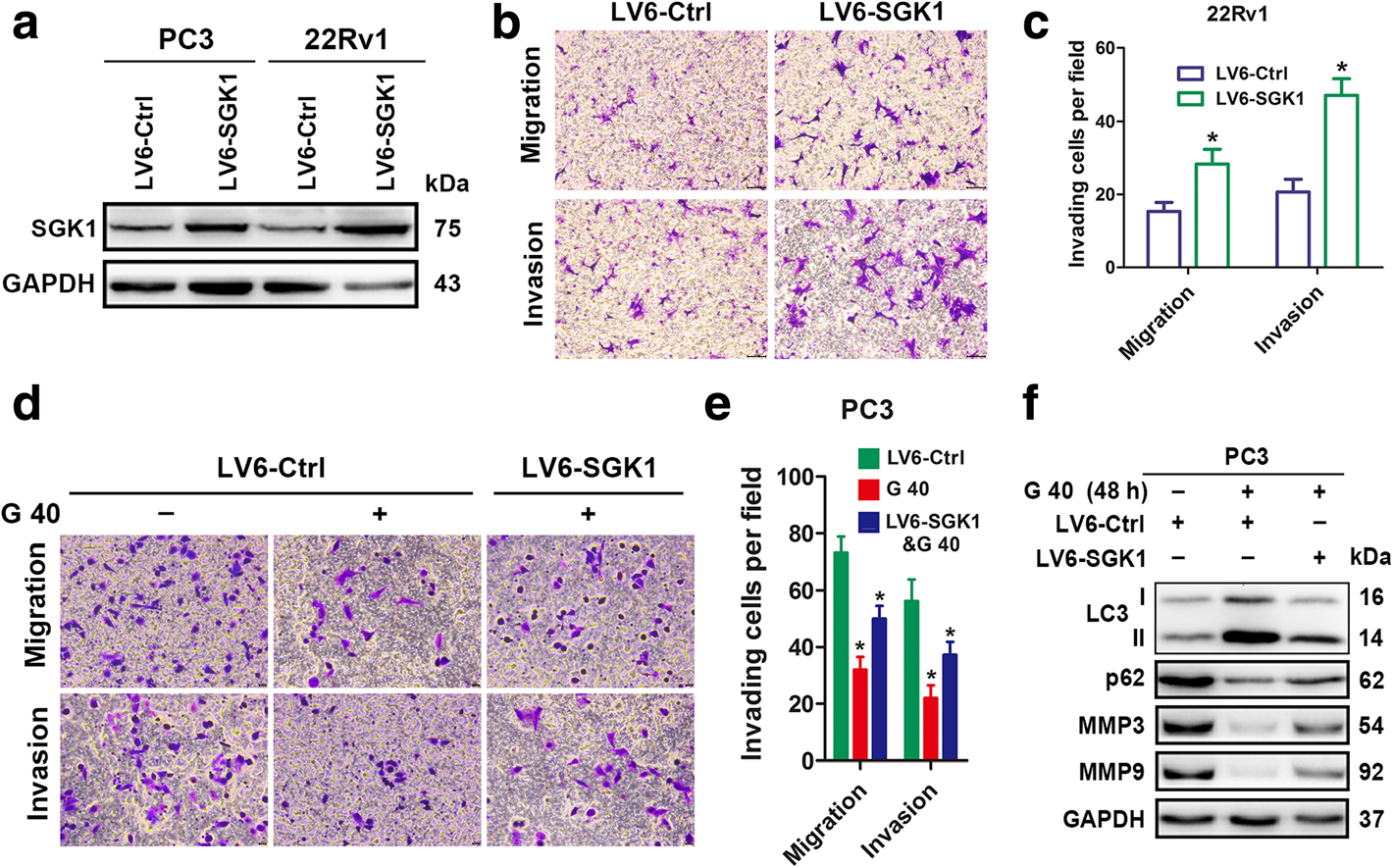
SGK1 overexpression attenuates autophagy and promotes cell migration and invasion. **a-c** PC3 and 22Rv1cells were infected with lentivirus containing an SGK1 (LV6-SGK1) expression plasmid or an empty vector (LV6-Ctrl) that confers resistance to puromycin. PC3 and 22Rv1cells cells stably overexpressing SGK1 or vector alone were assessed using a western blot against SGK1 (**a**), 6 × 10^4^ 22Rv1 for migration, 3 × 10^5^ 22Rv1 for invasion (**b**). Representative histograph of invaded tumor cells is displayed and number of invaded tumor cells quantified (**c**). **d-f** PC3 cells stably overexpressing SGK1 or vector alone were treated for 24 h with 40 μM GSK650394. Cell migration assays and matrigel cell invasion assays were performed. Representative fields of migration and invasion cells on the membrane are shown (magnifications, × 200) (**d**). And number of invaded tumor cells per field is quantified (**e**). Total protein lysates were collected after 48 h treatment with 40 μM GSK650394 for western blot against LC3, p62, MMP3 and MMP9, and GAPDH was used as a loading control (**f**). All results are representative of three experiments and are expressed as the mean ± S.D.; *P < 0.05

In order to further decipher the contribution of SGK1 in regulating metastasis, we overexpressed SGK1 in PC3 and 22Rv1 cells. Cells infected with lentivirus containing an SGK1 (LV6-SGK1) expression plasmid were shown to robustly increase the protein level of SGK1 compared to an empty vector (LV6-Ctrl) (Fig. 4a), which resulted in significant enhancement of cell migration and invasion capability both in PC3 and 22Rv1 cells (Fig. 4b-e). Next, PC3 cells stably overexpressing SGK1 or vector alone were treated with 40 μM GSK650394 for 24 h. Interestingly, overexpression of SGK1 was found to attenuate the autophagy and antagonize the inhibitory effects on cell metastasis induced by GSK650394 (Fig. 4d and e), which was further confirmed by the levels of LC3 conversion, p62, MMP3 and MMP9 (Fig. 4f). Thus, a direct role of SGK1 in the modulation of autophagy was demonstrated, and these data imply that SGK1 promotes cell metastasis, in part by attenuating autophagic activity.

### SGK1 inhibition-induced autophagy suppresses EMT through the downregulation of snail

**Fig. 5:**
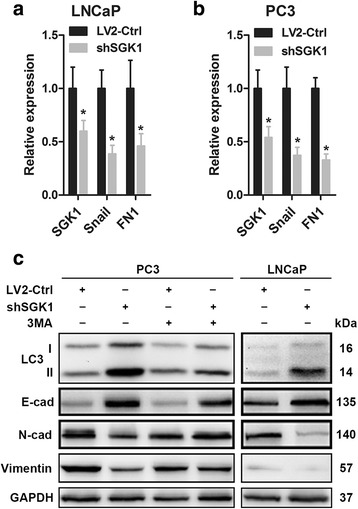
SGK1 inhibition-induced autophagy activates MET. (**a-b**) Quantitative RT-PCR analysis of SGK1, Snail, and Fibronectin (FN1) mRNA expression was performed in SGK1-depleted PC3 and LNCaP cells (shSGK1) and in control cells (LV2-Ctrl). (**c**) Effect of SGK1 inhibition-induced autophagy on EMT regulators. PC3 and LNCaP cells were infected with empty lentivirus control (LV2-Ctrl) or with the lentivirus SGK1-shRNA (LV2-shSGK1) and subsequently incubated for 48 h before pretreatment with 5 mM 3MA or PBS (control). Western blots were probed for levels of LC3, E-cad, N-cad and Vimentin, and GAPDH was used as a loading control. All results are representative of three experiments and are expressed as the mean ± S.D.; *P < 0.05

In order to gain an insight into the molecular mechanisms involved in SGK1 inhibition-induced autophagy-mediated regulation of cell migration and invasion, an analysis of the main EMT/MET players was performed. As shown in Fig. 5a and b, SGK1 silencing dramatically downregulates Snail and Fibronectin (FN1) mRNA levels both in PC3 and LNCaP cells compared to control cells, implying that SGK1 inhibition reverses EMT, at least partially through the downregulation of Snail expression. Several biochemical markers are used to characterize EMT: epithelial cells predominantly express E-cadherin, while mesenchymal cells express N-cadherin and Vimentin [22]. Since SGK1 inhibition-induced autophagy contributes to metastasis suppression, we determined whether SGK1 inhibition-induced autophagy is sufficient to inhibit EMT by examining expression of the aforementioned markers. Stable knockdown of SGK1 increased E-cadherin levels, while decreasing N-cadherin and Vimentin levels in both PC3 and LNCaP cell lines (Fig. 5c). Interestingly, our further results showed that 3MA-mediated autophagy inhibition dramaticlly reversed alterations of all the aforementioned markers induced by SGK1 silencing (Fig. 5c). Taken together, these results suggest that SGK1 inhibition-induced autophagy suppresses EMT, at least partially through the downregulation of Snail.

### Dual inhibition of mTOR and SGK1 enhances autophagy and leads to synergistic antimetastatic effects on PCa cells

**Fig. 6:**
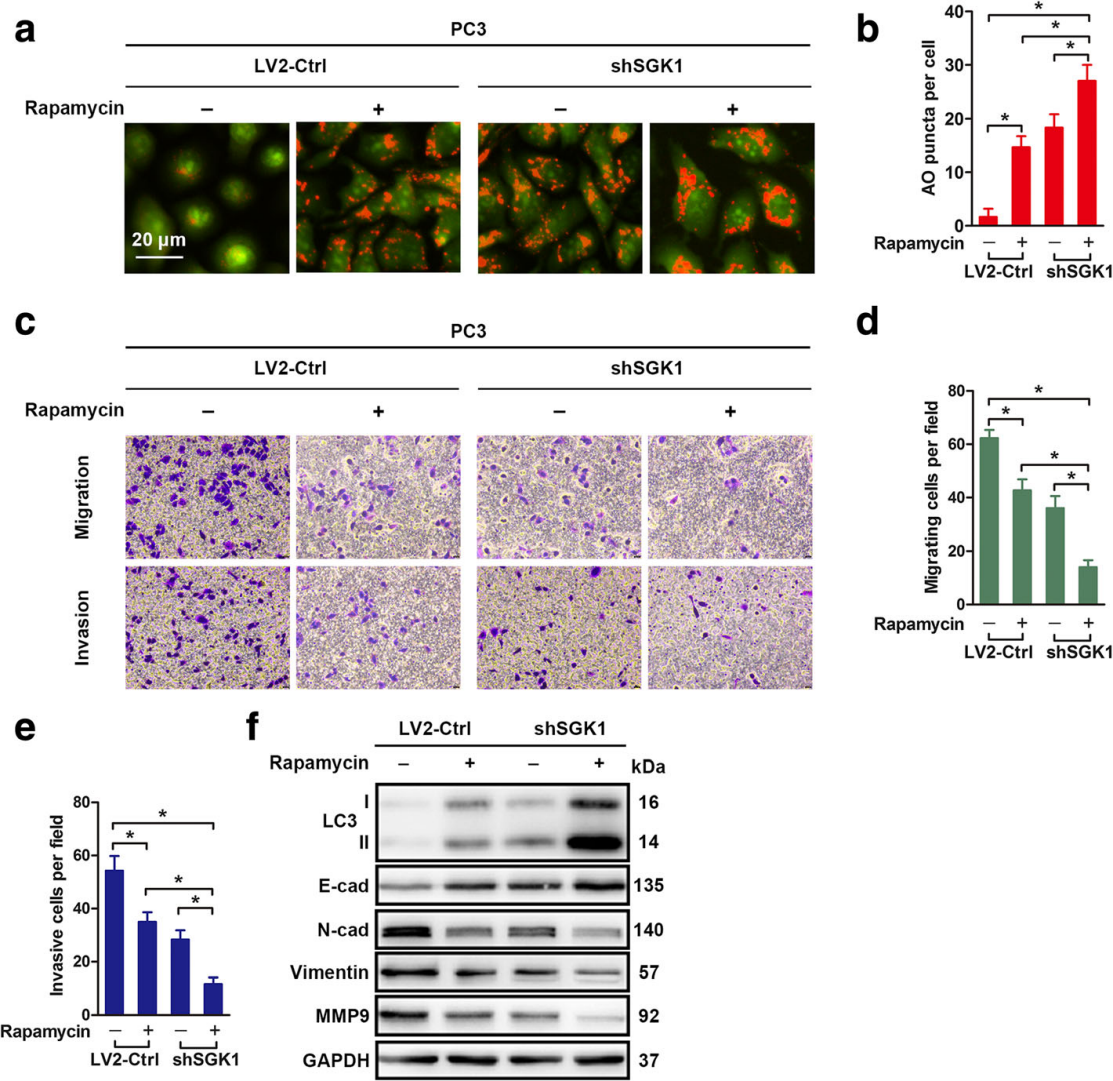
Dual inhibition of mTOR and SGK1 enhances autophagy and leads to synergistic antimetastatic effects on PCa cells. **a-f** PC3 cells were treated with DMSO or Rapamycin (100 nM) or infected with shSGK1 or a combination of both treatments for 24 h. Autophagy activity was determined with AO staining (**a**) and quantified (**b**). Cell migration assays and matrigel cell invasion assays were performed. Representative fields of migration and invasion cells on the membrane are shown (magnifications, × 200) (**c**). And number of migrating and invasive cells per field are quantified (**d-e**). **f** PC3 cells were treated as in (**a**) for 48 h. Total protein lysates of PC3 cells were assessed using western blotting against the indicated antigens. All results are representative of three experiments and expressed as the mean ± S.D.; *P < 0.05

Our results demonstrated that SGK1 inhibition-induced autophagy impaired cell metastasis. However, whether co-targeting SGK1 and autophagy could lead to more deleterious effect on PCa cell migration and invasion needs to be further investigated. Given the importance of mammalian target of rapamycin (mTOR) in autophagy modulation [22], thus rapamycin was used to activate autophagy. First, we examined the combination of rapamycin and shSGK1 on the induction of autophagy in PC3 cells using AO staining. As shown in Fig. 6a, treatment with either rapamycin or SGK1-silencing shRNA significantly increased AO puncta compared to vehicle control, and the combination of both exhibited a synergistic effect, with approximately 80% of the cells exhibiting more AO puncta than were observed with the individual treatments (Fig. 6b), which was further verified via LC3-I/LC3-II conversion (Fig. 6f). Then, the metastasis capability of PC3 cells was determined. Interestingly, while the impairment of cell migration and invasion upon rapamycin or SGK1 silencing were modest, the combination of both led to a more pronounced response than either treatment alone when compared with the control in PC3 cells (Fig. 6c-d). Moreover, it was found that the combined treatments significantly increased E-cadherin protein level compared to the individual treatments (Fig. 6f). Conversely, the combination of both dramaticlly decreased N-cadherin, Vimentin and MMP9 protein levels compared to either treatment alone (Fig. 6f). All in all, these results demonstrate that combined suppression of SGK1 and mTOR significantly increases autophagy activity and leads to synergistic antimetastatic effects on PCa cells. Therefore, the findings presented here add significant clues for the definition of future therapies targeting autophagy combined with other therapeutic strategies as means to suppress PCa metastasis.

### Downregulation of SGK1 inhibits metastasis in vivo

**Fig. 7:**
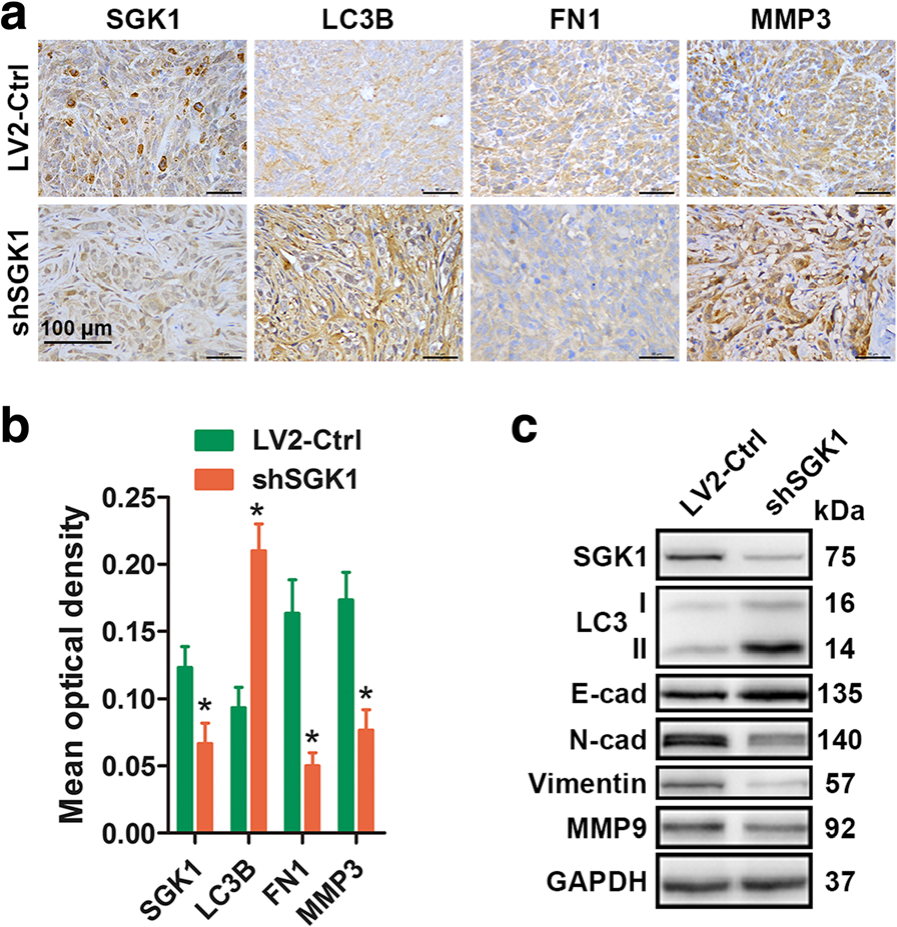
Downregulation of SGK1 inhibits metastasis in vivo. Briefly, 2 × 10^6^ PC3^LV2-Ctrl^ cells or 2 × 10^6^ PC3^shSGK1^ cells suspended in 0.2 ml PBS were inoculated subcutaneously in the right flank of each mouse. When the tumors of the PC3^LV2^ group reached approximately 500 mm^3^, all mice were sacrifice to collect the tumors. **a** SGK1, LC3B, Fibronectin (FN1) and MMP3 levels were determined by IHC. Representative images are presented. **b** Mean optical density of SGK1, LC3B, Fibronectin (FN1) and MMP3 was quantified by Image Pro-Plus. **c** The levels of SGK1, LC3B, E-cad, N-cad, Vimentin and MMP9 in tumor xenograft tissues were also detected by western blot. *P < 0.05

We next expanded our findings in vivo. The in vivo antimetastatic effects of SGK1 inhibition in PCa was determined in a tumor-transplant mouse model. It was found that there was a significant (80%) reduction in tumor weight in mice inoculated with PC3^shSGK1^ cells when compared with LV2-Ctrl mice (data shown in our previous study [20]). Immunohistochemistry demonstrated that SGK1, FN1 and MMP3 were downregulated, whereas LC3 was upregulated in the shSGK1 group compared to the LV2-Ctrl group (Fig. 7a and b). Immunoblotting results further confirmed that shSGK1 resulted in inhibition of SGK1, an increase in LC3-I/LC3-II conversion and E-cadherin, and an decrease in N-cadherin, Vimentin and MMP9 in vivo (Fig. 7c). On the basis of the above mentioned results, we conclud that SGK1 inhibition suppresses PCa metastasis at least partially via induction of autophagy and supression of EMT in vivo.

**Fig. 8:**
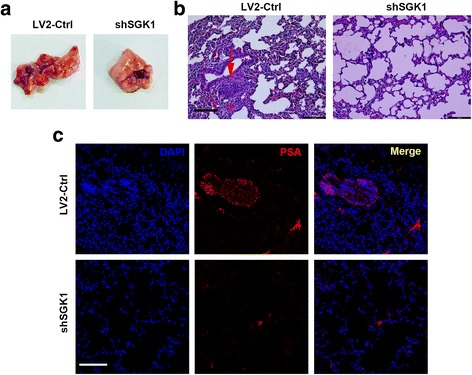
Downregulation of SGK1 inhibits metastasis in vivo. Briefly, mice were injected via tail vein with 1 × 10^6^ PC3^LV2-Ctrl^ cells or 1 × 10^6^ PC3^shSGK1^ cells suspended in 0.1 ml PBS. Mice were euthanized at 8 weeks post-implantation, lungs were obtained and shown (**a**). **b** Lungs were fixed in 10% formal dehyde, and hematoxylin and eosin (HE) stained for metastatic nodules, red arrow indicates metastatic nodule. (**c**) Representative immunofluorescence images of PSA expression in lung tissue. Representative images are presented, scale bar, 100 μm

A tail vein metastatic assay was further used to analyze the in vivo effects of SGK1 silencing on the metastatic potency of PC3 cells. Compared to the cells infected with shSGK1 virus vector, more nodules were observed in cells infected with empty virus vector in the lung (Fig. 8a). Wha’s more, HE staining showed that tail vein injection of cells stably infected with LV2-Ctrl into athymic nude mice led to significantly more and bigger nodules in the lung (Fig. 8b). To further confirm this distant metastasis, we performed immunofluorescence assay to detect critical markers of prostate cancer. Prostate specific antigen (PSA) was considered to be critical markers for diagnosis of prostate cancer. Thus, we used PSA in our study to investigate the source of tumor nodes in lung. As is shown in Fig. 8c, PSA positive in lung tissue indicated that the distant metastasis in the animal model was from the PC3 cells which were injected from tail vessel. These results showed the remarkable inhibitory effects of SGK1 silencing on pulmonary metastasis of prostate cancer cells.

## Discussion

Here we demonstrated that SGK1, a common downstream mediator of androgen receptor (AR) and glucocorticoid receptor (GR) signaling pathways [17], which displayed a significant upregulation expression in primary metastatic PCa tissues, could act as a tumor metastasis promoting gene in prostate cancer, as evidenced by its downregulation could suppress PCa cell lines invasion and migration capacity, whereas its overexpression could promote metastasis. Its elevated expression in primary metastatic PCa tissues indicates that SGK1 may be useful for prognosis and as being a positive predictor for relapse and metastasis of PCa. We further found that SGK1 inhibition-induced metastasis suppression function is dependent on its negative regulation on the EMT, at least partially through autophagy induction, as its deletion could confer resistance to Snail-induced EMT but in contrast its overexpression could attenuate autophagic activity and promote the EMT and metastasis in PCa.

This study highlights a novel and pivotal role for SGK1 in tumor metastasis. Although the role and function of SGK1 were extensively studied in cancer, mainly focusing on tumor growth (e.g., cell proliferation, apoptosis and cell cycle arrest) [23–27], little is known about the potential role of SGK1 in cancer metastasis. Our results showed that downregulation of SGK1 could significantly impair PCa invasion and migration capability in vitro and in vivo, whereas its overexpression could dramaticlly promote PCa metastasis. This invasion and metastasis-promoting function as characterized here in PCa cells are similar to that as shown previously in HEK293 cells [18]. In addition, Zarrinpashneh et al. [19] also found that ablation of SGK1 could impair endothelial cell migration and tube formation leading to decreased neo-angiogenesis following myocardial infarction. To some extent, these two previous studies further support our findings that SGK1 does play an important role in tumor invasion and migration.

As mentioned in the introduction, the EMT is widely considered to be an important mechanism regulating the initial steps in cancer metastasis. Owing to the clinical importance of this process, repression of EMT is an attractive therapeutic strategy that can significantly improve disease outcome. However, it remains unknown which pathways should be inhibited in order to reverse EMT. Our findings implicate SGK1 as a key regulator of EMT in PCa. This conclusion is based on the results that silencing SGK1 induces a repertoire of biochemical (increased E-cadherin and decreased Snail, N-cadherin, Vimentin and Fibronectin) and functional (decreased MMP3 and MMP9 production, and decreased invasion and migration capacity) changes characteristic of MET. Consistent with our findings, a previous study found that the downstream serine/threonine kinase mTOR, which is known to be an important upstream regulator of SGK1 [28, 29] and plays a crucial role in promoting EMT via RhoA and Rac1 signaling in colorectal cancer (CRC) [30]. In addition, forkhead transcription factor FKHRL1, also called Foxo3a, is the downstream target of SGK1 and plays a proapoptotic role by inducing cell cycle arrest and apoptosis [31, 32]. Besides its proapoptotic function, in fact, Foxo3a was suggested to play a significant role in tumor metastasis [33], and downregulation of Foxo3a could dramaticlly induce EMT of tumor cells by upregulating Snail, which promoted tumor cells metastasis in vitro and in vivo [34]. These results suggest that inhibition of EMT may be an important mechanism underlying the attenuated metastasis of PCa noted upon inhibition of SGK1.

Next, we further found that autophagy induced by SGK1 inhibition suppressed EMT of PCa. This is the first time that SGK1 inhibition-induced autophagy was suggested to play a negative role in EMT regulation. Our results showed that silencing SGK1 could markedly induce autophagy, which was similar to a recent observation that SI113, a SGK1 inhibitor, could dramaticlly trigger autophagy in glioblastoma multiforme (GBM) [23]. Interestingly, our further results showed that 3MA-mediated autophagy inhibition significantly antagonized the inhibitory effects of SGK1 inhibition on cell migration and invasion capacity, and extensively inhibited the EMT induced by SGK1 silencing. What’s more, overexpression of SGK1 could obviously attenuate the autophagy and antagonize the inhibitory effects on cell metastasis induced by GSK650394. All these results suggest that SGK1 inhibition-induced autophagy does inhibit EMT in PCa. Consistent with our results, autophagy activation has been associated to repression of EMT via degrading Snail and Twist in breast cancer models [35]. On the contrary, it has been demonstrated that Cadherin-6 (CDH6) drives EMT and cancer metastasis by restraining autophagy in papillary thyroid carcinomas (PTCs) [36]. Therefore, the role of autophagy in tumor EMT and metastasis is still a matter of debate, autophagy is poised to serve both pro- and anti-metastatic roles depending on contextual demands [37]. Further work using in vivo models is critical to clarify the precise functions of autophagy at each step of metastasis and to develop novel therapeutic strategies to inhibit metastasis.

Treatments targeting autophagy combined with other therapeutic approachs have been studied extensively [38], and this combination treatments usually achieve better curative effects [39, 40]. In the present study, we found that mTOR inhibition-induced autophagy synergized with SGK1 inhibition could potently amplified the antimetastatic effects on PCa cells. Furthermore, the combined treatments significantly increased E-cadherin protein level and dramaticlly decreased N-cadherin, Vimentin and MMP9 protein levels compared to either treatment alone. Consistently, either inhibiting mTORC1 or mTORC2 is sufficient to induce MET and enhances sensitivity to oxaliplatin-induced apoptosis via RhoA and Rac1 signaling pathways in colorectal cancer cells [30]. In this regard, autophagy activation is just one of the mechanisms of mTOR inhibition-induced repression of EMT and supression of metastasis. In summary, co-targeting mTOR and SGK1 could significantly prevents PCa metastasis by reversing EMT due to the amplified antimetastatic effects.

In conclusion, the current study shows that SGK1 inhibition can suppress cell migration and invasion in PCa, at least partially via autophagy-mediated repression of EMT through the downregulation of Snail. Our study also demonstrates the functional significance of its upregulation in metastatic PCa, and presents a novel combination therapy regimen that co-targeting SGK1 and autophagy restrains cancer progression due to the amplified antimetastatic effects.

## Methods

### Cell lines and cell culture

PC3, LNCaP, DU145 and CWR22-Rv1 prostate cancer cell lines were purchased from American Type Culture Collection, cells were cultured in RPMI-1640 media supplemented with 10% fetal bovine serum (FBS) in 37 °C and 5% CO_2_ atmosphere.

### Reagents and antibodies

GSK650394, 3-methyladenine (3MA) and Rapamycin were purchased from Selleck. Acridine orange (AO) was obtained from Beyotime (Haimen, China). Antibodies against E-cadherin (#14472), N-cadherin (#13116), Vimentin (#5741), LC3B (#3868), and GAPDH (#5174) were purchased from Cell Signaling Technology (Beverl y, MA, USA). Antibodies against SGK1 (ab59337) and P62 (ab56416) were purchased from Abcam (Cambridge, USA). Antibodies against MMP3 (sc-6839) and MMP9 (sc-21,733) were purchased from Santa Cruz Biotechnology (Santa Cruz, CA). Secondary antibodies (HRP-conjugated sheep anti-rabbit antibodies or HRP-conjugated sheep anti-mouse antibodies) for western blot were obtained from Millipore (Shanghai, China).

### Wound healing assay

Wound healing assay was conducted to examine the capacity of cell migration. Briefly, the wound was generated when the cells reached 80–90% confluent in a six-well plate by scratching the surface with 10 μL pipette tip. The cells were then incubated in medium containing 2% FBS for 24 h and 48 h, and then photographed using phase-contrast microscopy (Leica). To analyze the cell migration, the wounded areas were photographed at the indicated time points and processed using. Percentage of wound healing was measured as following: [1 - (empty area X h/empty area 0 h)] × 100.

### Migration and invasion assay

Unless otherwise specified, cell migration and invasion assay were determined by using a modified two chamber migration assay with a pore size of 8 μm. For migration assay, 3 × 10^4^ PCa cells were seeded in serum-free medium in the upper chamber. After 24 h incubation at 37 °C, cells in the upper chamber were carefully removed with a cotton swab and the cells that had traversed the membrane were fixed in methanol, stained with hematoxylin and counted. For invasion assay, the matrigel (BD Biosciences, CA, USA) was added to each well according to manufacturer’s instructions before 2 × 10^5^ PCa cells were seeded on the upper chamber. After 24 h incubation at 37 °C, noinvasive cells were gently removed from the top of the matrigel with a cotton-tipped swab. Invasive cells at the bottom of the matrigel were fixed in methanol and stained with leucocrystal violet. For quantification, cells were counted under a microscope in five fields (up, down, median, left, right. × 200).

### Immunohistochemistry and evaluation of immunostaining

Formalin-fixed paraffin-embedded (FFPE) tissue samples from PCa and corresponding adjacent normal prostate tissues (at least 1.5 cm away from the tumor) were collected from 30 patients who were histopathologically diagnosed with PCa and underwent surgical treatment at the Second Affiliated Hospital of the Medical College of Zhejiang University (Zhejiang, China) during the past 2 years. Briefly, 4-μm-thick sections mounted on glass slides were processed for immunohistochemistry (IHC). All slides were dewaxed in xylene and dehydrated in an alcohol gradient, and then, endogenous peroxidase activity was quenched with 3% hydrogen peroxide for 10 min. Antigen retrieval was achieved by heating slides covered with citrate buffer (pH = 6.0) at 95 °C for 10 min. Then, 10% goat serum albumin was used to block nonspecific binding by incubating sections for 2 h at room temperature while gently tilting the sections without washing them, followed by incubation with SGK1, Foxo3a, p-Foxo3a, mTOR, LC3B and p62 antibodies at 4 °C overnight in a moist chamber. After being washed three times with PBS, the sections were incubated with a secondary antibody at room temperature for 1 h and rinsed in phosphate-buffered saline (PBS). Diaminobenzidine (DAB) was used as a chromogen, and sections were counterstained with hematoxylin. Negative controls were obtained by incubating specimens with PBS instead of primary antibody. Brown particles present in the cytoplasm and/or nuclei were considered positive signals. Any nuclear and/or cytoplasmic immunoreactivity was recorded. Intensity was graded on a three-point scale from 1 to 3 to represent low, intermediate and high expression, respectively. This scoring was performed by two pathologists, who reviewed the immunoreactivity together and arrived at a consensus score that was used in the analyses.

### Cell proliferation assay

Cell suspension of 100 μl was dispensed (4–6 × 10^3^ cells/well) in 96-well plates. Cell proliferation was measured using a Cell Counting Kit-8 (CCK-8, Dojindo Molecular Technologies, Kumamoto, Japan) following the manufacturer’s instructions with plate incubation for 2 h. The absorbance was measured at 450 nm using a micro-plate spectrophotometer (BIO-RAD xMark).

### Vital staining with AO

PCa cells were seeded in a 24-well culture plate and after subsequent treatments, cells were stained with AO (1 μg/mL) directly for 10 min at 37 °C, and then observed under the fluorescence microscope (Olympus, Tokyo, Japan).

### Transfection with lentiviral particles

To generate SGK1-silencing and SGK1-overexpressing stable PCa cell lines, PCa cells were infected with lentiviral particles. The lentiviral expression vectors LV2-Control, LV2-shSGK1, LV6-Control and LV6-SGK1 (SGK1-overexpression) were purchased from Shanghai Gene Pharma Company (China). In brief, the cells were seeded at 2 × 10^5^ cells/well in a 6-well plate before lentiviral particle infection and incubated with 2 ml of complete medium for 24 h. Then, cells were infected with lentiviral particles, and after 12 h, the virus-containing medium of infected cells was substituted with fresh complete medium, and infected cells were selected with 4 μg/ml puromycin for 96 h. Empty lentiviral vectors were used as a control.

### Quantitative real-time PCR

Total RNA was isolated from cells using TRIzol reagent (Takara, Dalian, China) and reversely transcribed through PrimeScript RT-PCR kit (TaKaRa Biotechnology) according to the protocol. The qRT-PCR was performed on the ABI 7900 Prism HT (Applied Biosystems), followed by melting curve analysis. The 2^-△△Ct^ method was used to assess the gene expression levels. Primers used are followed: SGK1, Forward: AGGATGGGTCTGAACGACTTT; Reverse: GCCCTTTCCGATCACTTTCAAG; Snail, Forward: TCGGAAGCCTAACTACAGCGA; Reverse: AGATGAGCATTGGCAGCGAG; Fibronectin, Forward: ACCCTCACCAACCTCACTC; Reverse: CCTCGGAACATCAGAAACT; GAPDH, Forward: AACAGCCTCAAGATCATCAGCA; Reverse: CATGAGTCCTTCCACGATACCA.

### Western blot analysis

PCa cells were seeded at a density of 5 × 10^5^ cells per well in a 6-well culture plate and harvested after subsequent treatments, whole protein lysates were collected cells using RIPA cell lysis reagent containing proteinase and phosphatase inhibitors (Solarbio) at 4 °C for 30 min. Cell lysates were centrifuged at 12,000 g for 20 min at 4 °C, and Protein concentrations of the supernatants were determined using the BCA protein assay reagent kit (Beyotime, Shanghai, China). Then, the supernatants containing total proteins were mixed with corresponding volume of 5 × SDS loading buffer and heated at 95 °C for 5 min, and 40 μg of total protein from each sample were concentrated on 5% Tris–Glycine SDS gels, separated on 8% or 12% Tris–Glycine SDS gels, and transferred to 0.22-μm polyvinylidene fluoride (PVDF) membranes. The membranes were blocked with 5% non-fat dry milk in TBST, incubated overnight with the appropriate primary antibody (1:1000). After being washed three times for 30 min with TBST, the membrane was incubated with HRP-conjugated secondary antibodies (1:5000) for 2 h at room temperature. The bound antibodies were visualized using an enhanced chemiluminescence kit (Millipore, Billerica, MA, USA).

### In vivo tumor biology

Animal studies were conducted in accordance with institutional ethical guidelines for the care and use of experimental animals. Briefly, 4-week-old female BALB/c-nu mice were purchased from Shanghai Laboratory Animal Center of the Chinese Academy of Sciences. They were maintained under specific pathogen-free conditions and supplied with sterilized food and water. For in vivo xenograft studies, ten mice were randomly selected and divided into two groups. On day 0, 2 × 10^6^ PC3^LV2-Ctrl^ cells or 2 × 10^6^ PC3^shSGK1^ cells suspended in 0.2 ml of PBS were inoculated subcutaneously in the right flank of each mouse (five mice in each group). Tumor sizes were measured daily to observe dynamic changes in tumor growth and calculated by a standard formula, length × width × depth × 0.5236. Tumor formation was defined as the time from inoculation until tumors measured 100 mm^3^. Subsequently, tumor volume measurements were performed twice weekly, and when the tumors of the PC3^LV2^ group reached approximately 500 mm^3^, all mice were killed. Tumors were dissected and stored in liquid nitrogen or fixed in formalin for further analysis. All treatment protocols were approved by the Animal Care and Use Committee of Zhejiang University, China.

### In vivo experimental metastasis models

Two groups of 10 male nude mice were housed in SPF barrier facilities under a 12 h light/dark cycle. Mice were injected via tail vein with 1 × 10^6^ PC3^LV2-Ctrl^ cells or 1 × 10^6^ PC3^shSGK1^ cells suspended in 0.1 ml PBS. Mice were euthanized at 8 weeks post-implantation, lungs were dissected and stored in liquid nitrogen or fixed in formalin for further analysis, and hematoxylin and eosin (HE) stained for metastatic nodules. Prostate specific antigen (PSA) was detected by immunofluorescence assay to investigate the source of tumor nodes in lung. All treatment protocols were approved by the Animal Care and Use Committee of Zhejiang University, China.

### Statistical analysis

The values are shown as the means±S.D. for triplicate experiments, and significant differences were calculated using one-way ANOVA with Dunnett’s test or Newman-Keuls test and Student’s two-tailed t-test. Levels of statistical significance were set at *P* < 0.05 or 0.01 as indicated.

## Additional file

Additional file 1: **Figure S1.** GSK650394 impairs DU145 cells migration capability.Wound healing assays of DU145 cells treated with DMSO or 20 μM GSK650394. Phase-contrast images were acquired at 0 and 24 h after scratching and representative images of three independent experiments are shown. The wound healing area was analyzed by using ImageJ software and the corresponding data, relative to 0 h, expressed in the graph. (DOCX 961 kb)

## Abbreviations

3-methyladenine: 3MA
Acridine orange: AO
Adjacent normal prostate tissues: AT
Androgen receptor: AR
Cadherin-6: CDH6
Cell counting Kit-8: CCK-8
Diaminobenzidine: DAB
Epithelial-mesenchymal transition: EMT
Fibronectin: FN1
Formalin-fixed paraffin-embedded: FFPE
Glucocorticoid receptor: GR
Hematoxylin and eosin: HE
Immunohistochemistry: IHC
Mammalian target of rapamycin: mTOR
Mesenchymal-epithelial transition: MET
Metastatic PCa: mPCa
Nonmetastatic PCa: NmPCa
Phosphate-buffered saline: PBS
Prostate cancer: PCa
Prostate specific antigen: PSA
Protein kinase B: PKB
Serum- and glucocorticoid-induced protein kinase 1: SGK1
