# Supplementary material for: SGK1 inhibition-induced autophagy impairs prostate cancer metastasis by reversing EMT
Source: J Exp Clin Cancer Res. 2018 Apr 2;37:73. doi: 10.1186/s13046-018-0743-1 (PMC5879613; doi:10.1186/s13046-018-0743-1)
Supplement: Supplementary file 1 — Figure S1. GSK650394 impairs DU145 cells migration capability.Wound healing assays of DU145 cells treated with DMSO or 20 μM GSK650394. Phase-contrast images were acquired at 0 and 24 h after scratching and representative images of three independent experiments are shown. The wound healing area was analyzed by using ImageJ software and the corresponding data, relative to 0 h, expressed in the graph. (DOCX 961 kb) [file 13046_2018_743_MOESM1_ESM.docx]

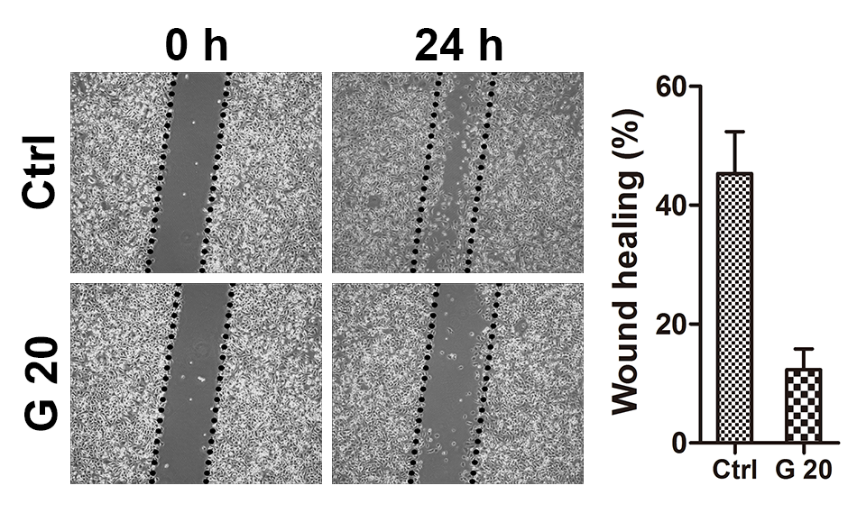


**Supplementary Figure 1 | GSK650394 impairs DU145 cells migration capability.**

Wound healing assays of DU145 cells treated with DMSO or 20 μM GSK650394. Phase-contrast images were acquired at 0 and 24 h after scratching and representative images of three independent experiments are shown. The wound healing area was analyzed by using ImageJ software and the corresponding data, relative to 0 h, expressed in the graph.
